# Developing and Evaluating a Flexible Wireless Microcoil Array Based Integrated Interface for Epidural Cortical Stimulation

**DOI:** 10.3390/ijms18020335

**Published:** 2017-02-05

**Authors:** Xing Wang, Sharjeel A. Chaudhry, Wensheng Hou, Xiaofeng Jia

**Affiliations:** 1 Key Laboratory of Biorheological Science and Technology, Ministry of Education, College of Bioengineering, Chongqing University, Chongqing 400044, China; wangxing@cqu.edu.cn (X.W.); w.s.hou@cqu.edu.cn (W.H.); 2 School of Medicine and Health Sciences, George Washington University, Washington, DC 20037, USA; sharjeel@gwu.edu; 3 Department of Neurosurgery, University of Maryland School of Medicine, Baltimore, MD 21201, USA; 4 Department of Orthopaedics, University of Maryland School of Medicine, Baltimore, MD 21201, USA; 5 Department of Anatomy and Neurobiology, University of Maryland School of Medicine, Baltimore, MD 21201, USA; 6 Department of Biomedical Engineering, Johns Hopkins University School of Medicine, Baltimore, MD 21205, USA; 7 Department of Anesthesiology & Critical Care Medicine, Johns Hopkins University School of Medicine, Baltimore, MD 21205, USA

**Keywords:** stroke, rehabilitation, inductive coupling, micro coil, nerve-electrode interface, epidural cortical stimulation

## Abstract

Stroke leads to serious long-term disability. Electrical epidural cortical stimulation has made significant improvements in stroke rehabilitation therapy. We developed a preliminary wireless implantable passive interface, which consists of a stimulating surface electrode, receiving coil, and single flexible passive demodulated circuit printed by flexible printed circuit (FPC) technique and output pulse voltage stimulus by inductively coupling an external circuit. The wireless implantable board was implanted in cats’ unilateral epidural space for electrical stimulation of the primary visual cortex (V1) while the evoked responses were recorded on the contralateral V1 using a needle electrode. The wireless implantable board output stable monophasic voltage stimuli. The amplitude of the monophasic voltage output could be adjusted by controlling the voltage of the transmitter circuit within a range of 5–20 V. In acute experiment, cortico-cortical evoked potential (CCEP) response was recorded on the contralateral V1. The amplitude of N2 in CCEP was modulated by adjusting the stimulation intensity of the wireless interface. These results demonstrated that a wireless interface based on a microcoil array can offer a valuable tool for researchers to explore electrical stimulation in research and the dura mater-electrode interface can effectively transmit electrical stimulation.

## 1. Introduction

Stroke remains a leading cause of serious long-term disability [1]. Survivors routinely have incomplete cognitive recovery and chronic motor and visual impairments that hinder their rehabilitation [2,3,4]. Recent evidence indicates that limb rehabilitation, which focuses on helping patients achieve clinically meaningful improvements in motor control, facilitates motor relearning for stroke survivors [4], but the majority of patients do not regain adequate motor function with rehabilitation alone. Neural plasticity, which is presumed to play a major role in motor recovery, can be modulated by various exogenous methods, including electrical stimulation [5,6]. Epidural cortical stimulation (ECS), a therapeutic that exposes dura mater by craniotomy for electrical stimulation of the cortex, has demonstrated significant improvements in motor function in animal models of stroke and as an adjunctive treatment in limb motor function rehabilitation of stroke patients with hemiparesis by modulating neuronal plasticity [4,7,8,9,10,11,12]. In addition, approximately one-third of stroke patients present with the devastating consequences of aphasia, the inability to speak [13]. Phase I Clinical Trials reported by Cherney have confirmed ECS as an adjuvant intervention for chronic nonfluent aphasia patients [13]. Moreover, approximately 60% of stroke survivors report visual impairment. Human trials of visual cortex electrical stimulation with both surface and penetrating electrodes have demonstrated effectiveness in improving visual ability [14], while limited commercial success has been achieved [15]. Flexible surface electrodes have demonstrated safety and efficiency in chronic ECS of stroke survivors, including flexible electrode and grid electrode arrays [13].

Although implantable pulse generators (IPGs) have shown precise stimulation and therapeutic effects [16,17,18,19], there is a critical need for a compact IPG to further explore ECS in research settings. Currently used IPGs for ECS encounter device-related adverse effects including lead failure [17], lead migration (13% to 30%), lead breakage (5% to 10%), infection (1% to 20%), and pain at the implant site (5% to 20%) [4]. Infections can also occur due to IPG batteries [20]. Limiting device-associated outcomes will broaden the usability of ECS in research and clinical trials. Despite its pitfalls, ECS reduces the degree of invasiveness and lowers the infection risk when compared to direct cortical stimulation systems [21].

Current systems used in ECS also face key challenges associated with their miniaturization and electrode flexibility [22]. Nerve electrodes come with a trade-off between invasiveness and selectivity [23]. The senior author has used intrafascicular electrodes [24,25] and developed bio-degradable regenerative multiluminal conduits with better precision during neural recording [26], while mainly for peripheral nerve interface. The Hou Team has designed surface electrode arrays using a flexible printed circuit (FPC) technique for primary visual cortex stimulation [27], for its softness can accommodate tissue micromotion and the delicate and convoluted curvature of the cortex within the skull [28]. However, the multiple wire connections between the electrode and stimulator circuit were vulnerable and easy to twist, which could possibly disrupt the IPG [29], thus this system was not feasible for ECS. Recently, Mou and Hou et al. explored a multichannel signal transmission system using a microcoil array [30]. Due to poor output controllability of the signal generator inside the transmitter, this system could not be used for electrical stimulation.

In this paper, we present an improved single-channel IPG prototype that receives pulse voltage stimuli from an outside transmitter and outputs the stimuli to a stimulating electrode. In this paper, we present an improved single-channel IPG prototype that receives pulse voltage stimuli from an outside transmitter and outputs the stimuli to a stimulating electrode. While previous studies have used wired stimulation devices for ECS, this study examines an implantable neural interface that is fabricated wholly on one piece of soft FPC board with passive circuit. This technique may help overcome many clinical challenges associated with IPG implants through its portable-size, wireless power, and integrated scheme. This study also indicates the feasibility of activating neurons by ECS in the primary visual cortex using a passive wireless IPG fabricated by FPC, making these early results a promising indicator towards modulating neuronal excitation in neuro-rehabilitation of stroke patients with vision loss.

## 2. Results

### 2.1. Benchtop Testing

The transmitting coil of a PCB coil with the diameter of 8.4 mm at 1.8432 MHz frequency had an inductance of 2.27 µH, a resistance of 8.09 Ω and a *Q* value of 3.24. The receiving coil of a four-layer FPC coil with diameter of 5 mm at 1.8432 MHz frequency had an inductance of 17.27 µH, a resistance of 50.56 Ω and a *Q* value of 4.29. At axial coil distance of 3 mm, when horizontal mismatch was within 3 mm and angular mismatch was within 20°, the output voltage amplitude would decrease 15%. In the implantable passive board, the receiving coil of the four-layer FPC spinal coil had an inductance of 8.45 µH, resistance of 27.27 Ω, and *Q* of 3.59 at 1.8 MHz frequency. The impedance of the flexible surface stimulating electrode ranged from 500–800 Ω at the testing frequency of 1 KHz using a three-electrode setup test system. As seen in Table 1, *V*_o_ increased when *V*_pc_ was raised. The relationship between *V*_o_ and *V*_pc_ was not linear. The output current decreased when the load resistance increased. The output results at 1 and 2 Hz were similar to those at 0.5 Hz. In the test of the two-channel system, the coupling voltage was 8.741 V at the first coil, the noise was −0.3479 V at 2 MHz carrier, and the input current was 1 A in both channels.

### 2.2. In Vivo Study

**Figure 1:**
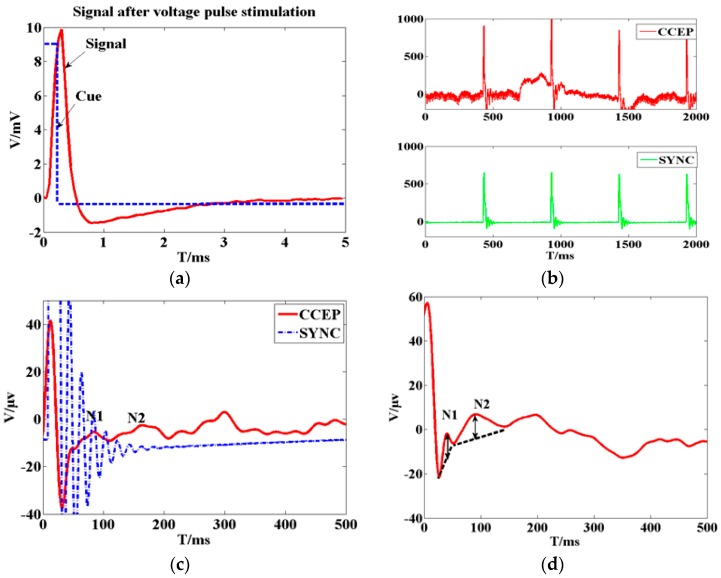
(**a**) The recording voltage in primary visual cortex by glass tungsten electrode when sub-threshold voltage pulse stimulus was delivered on the ipsilateral dura mater by external wired stimulator device. The blue dash line shows the square pulse stimulus with 200 µs pulse width and 400 mV. The red line waveform shows the recording signal with peak voltage of 10 mV. The result shows that stimulus on the dura mater can be transferred to cortical tissue; (**b**) Original cortico-cortical evoked potential (CCEP) (in red color) recorded on unilateral visual cortex when voltage stimulus was delivered on the contralateral dura mater by the wireless stimulator device. The green trace is for stimulating synchronization signal that output from the external transmitter; (**c**) Cortical evoked potential after averaging and filtering; (**d**) Typical configuration of CCEPs.

Three out of five experiments provided successful data (one failed experiment had strong noise and the other had low quality physiological signals). The results in Figure 1a show that epidural pulse voltage stimulus can be transferred effectively to cortical tissue. An obvious striking peak appears in the cortico-cortical evoked potential (CCEP) signal in the beginning (Figure 1b), which was defined as the electrical artifact of the stimuli because the striking peak’s width was consistent with the synchronous signal. The lock time property between the evoked response and the stimuli synchronized signal verified that the implantable wireless stimulation system led to an effective stimulation.

A fragment of 500 ms length data was chosen for analysis to capture the general visual evoked potential (VEP) period of 300 ms. Figure 1c shows the typical configuration of cortico-cortical evoked potentials (CCEPs) CCEP typically consists of a negative surface deflection termed as N1, and a later (80–250 ms) slow wave as N2. CCEP latency and amplitude were analyzed by measuring the first two negative waves in Figure 1d. The electrical artifact was followed by a robust N1 peak, followed by N2 and N3. The second peak (N2) occurred within 200 ms. When stimulation intensity increased, the amplitude of N1 increased gradually, and the latency of N1 remained steady. This showed that the artifact changed when stimulation intensity increased. As seen in Figure 1c,d, the CCEP on the cat cortex appeared as an obvious capacitive charge/discharge phenomena because of cerebrospinal fuild’s contribution.

**Figure 2:**
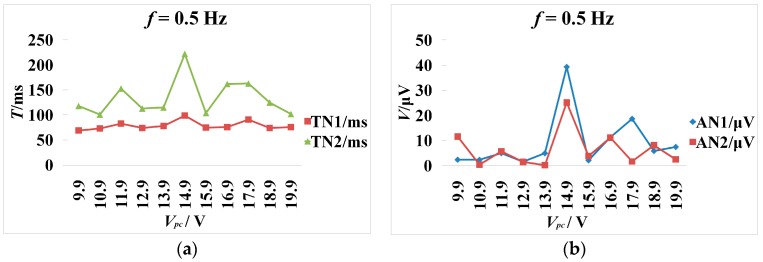
CCEPs change along with the varying stimulation intensity. The frequency is 0.5 Hz. *V*_oc_ = 5 V. *V*_pc_ = 9.9–19.9V. The peak-peak amplitude and offset of square voltage pulse are 3.3 and 1.65 V. The pulse width is 0.5 ms. (**a**) The latency change of N1 and N2 (negative surface deflections) along with increasing *V*_pc_. TN1 and TN2 represents the latency of N1 and N2; (**b**) The amplitude of N1 and N2 change along with the increasing *V*_pc_. AN1 and AN2 represent the amplitude of N1 and N2, respectively.

The CCEP features showed that *V*_pc_ regulated CCEPs in the cat’s visual cortex (V1). The threshold voltage was 6 V for *V*_pc_ at 0.5 Hz stimuli frequency because obvious evoked peaks and troughs appeared until *V*_pc_ was higher than 6 V (Figure 2a,b). When *V*_pc_ was increased to less than 14.9 V, the amplitude of the peak and trough also increased and latencies were shortened. The maximum amplitude of N1 and N2 took place when *V*_pc_ increased to 14.9 V. When *V*_pc_ increased to 15.9 V, the amplitude of N1 and N2 decreased, and the main trend seen was the repolarization wave of the stimulus artifact. When *V*_pc_ was continuously increased to 16.9 V, the sinusoid wave feature gradually weakened and the repolarization of the stimulus artifact was the main trend. N2 amplitude was about 0.256–27.269 µV at 0.5 Hz, and N1 latency was about 69–99 ms.

## 3. Discussion

In this paper, we present a single-channel IPG prototype that receives pulse voltage using inductive coupling from an external transmitter and delivers stimuli pulses to nerve tissue through a stimulating electrode. We employed an analog passive circuit scheme to build the system, which minimized the size of the IPG prototype circuit and did not need a power supply. Unlike the previously published iteration [30], which used a NE555 chip whose pulse output width and frequency could not be adjusted, our single-channel IPG has a two-stage signal generator circuit to regulate the width and frequency of the pulse voltage output. The first stage uses an ICL8038, which regulates the frequency of the output signal. The second stage regulates the pulse width using an NE555 chip. This two-step adjustment design improves the controllability and precision of the monophasic pulse voltage at the transmitter side.

While researchers work to fine-tune clinical protocols for implanting IPGs for ECS and using them as an adjuvant for stroke patients, our work presents novel improvements to the cortical stimulation system itself. We developed a small-size integrated stimulation interface, which employed a passive circuit, was fabricated wholly on a FPC board 12 mm × 8 mm in area, and provides a potential implant scheme for chronic cortical stimulation. Some commercially available IPGs have shown therapeutic effects as implantable cardioverter-defibrillators (ICDs) [16,17], spinal cord stimulation (SCS) IPGs [18] for Parkinson’s disease [19], and ECS therapy to improve motor function [31,32] and nonfluent aphasia [13] after stroke. However, because of their large size batteries, these IPGs are implanted in the chest area and wired electrodes are channeled through the human body to the stimulation site. This can cause lead failure and subsequent therapeutic stimulation outages [17]. Limiting the pocket size could reduce the incidence of Twiddler’s syndrome [29], which occurs due to coiling of the lead. Our small-size passive stimulation interface using inductive coupling could be implanted into a skull window directly near the stimulation target tissue. Since our integrated scheme does not have lead wires, it has the potential to alleviate lead failure and lead coiling. While our device uses inductive coupling for power, some emerging wireless powering technologies use ultrasonic neural dust (3 mm × 1 mm × 1 mm) [33], near-infrared light, body energy [34], and microwaves [35]. Battery-less wireless power is a growing trend in implant bioelectronics despite its challenges.

Another major challenge that bioelectronics interventions face is their lack of flexibility. Flexibility and softness are important because they determine if conformal contact with tissue can be made, which is responsible for affecting a minimal inflammatory response and leads to stable in dwellings over long periods of time [22]. Our whole system is fabricated into a small board and has extensibility, which prevents the interface from breaking down due to the accumulating stress of brain vessel impulses. Our FPC board produces a relatively soft circuit board and offers great flexibility and compliance.

**Figure 3:**
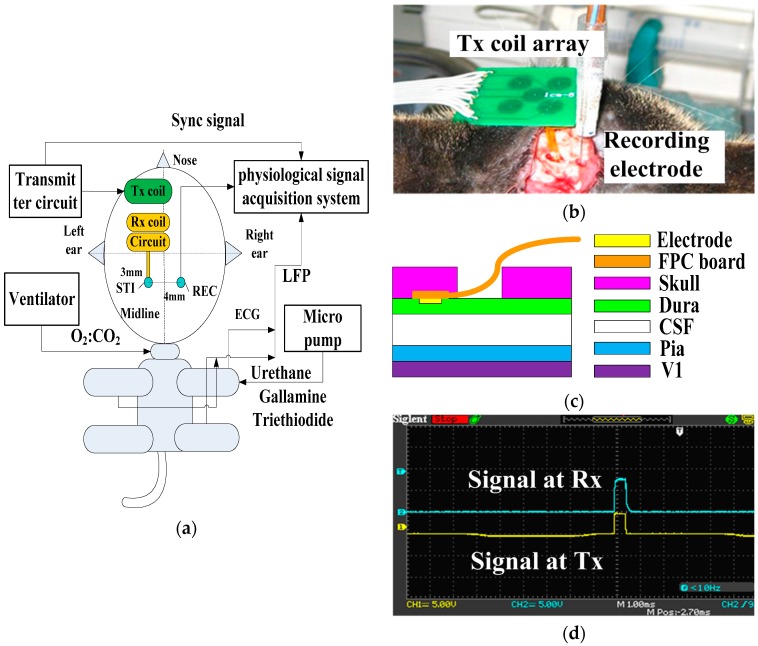

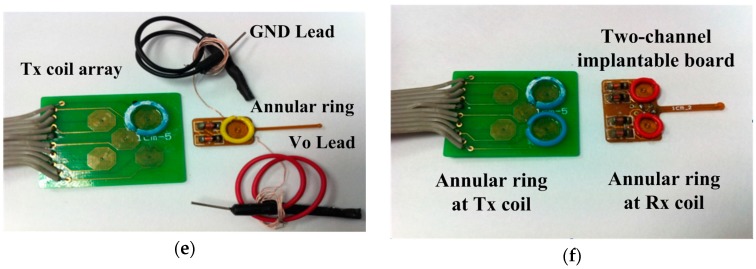
(**a**) The schematic diagram of the in vivo experimental set-up: the stimulating electrode and the recording electrode were located at the bilateral primary visual cortex. The implantable stimulator board acquired the voltage stimulus by inductively coupling with the external transmitter circuit. The neuronal responses were recorded by electrode and conveyed to the physiological signal acquisition system at the same time; (**b**) Photograph of the in vivo experiment: the implantable stimulator and recording electrode on the dura mater above primary visual cortex; (**c**) Cross-section schematic of electrical epidural stimulation; (**d**) The output stimulating voltage pulse signal (in blue color) at the implantable pulse generator (IPG) board. The yellow trace was input signal at external transmitter; (**e**) The transmitting coil array board with one annular ring (in blue color) and the single-channel implantable naked interface with two testing leads and an annular ring (in yellow color); (**f**) The transmitting coil array board with annular rings (in blue color) and the two-channel implantable board with annular rings (in red color) with 2 mm height.

Our work investigated the efficacy of the proposed IPG on the visual cortex using epidural electrical stimulation, potentially providing a novel method for sensation restoration in stroke survivors. Previous experimental work has been carried out using wired stimulation devices [36]. In order to increase the portability of the stimulator, we developed the wireless small-size pulse generator and demonstrated its feasibility in ECS. Despite the fact that the complicated electrode-tissue interface of epidural stimulation reduced the stimuli’s focusing capability and enlarged the actual stimulus coverage area, our epidural stimulation results from the cat visual cortex indicated that the neurons in the visual cortex could be activated by electric stimulation through the dura mater using an IPG. While most systems use direct stimulation, our electrode-neuron interface resulted in longer latencies possibly because it crossed more structures, namely the dura mater, CSF, pia mater, unilateral visual cortex, callosum, and contralateral visual cortex as shown in Figure 3c.

A major issue in wireless implants based on radio frequency is the stability of the output stimulation and external interference. Possible reasons for the non-linearity in Table 2 are micro-motion of the coil and the non-linearity of the circuit. The stability and accuracy of our system needs further improvement for chronic settings. One way this can be achieved is by fabricating an annular wall using a high-permeability material around the entire circuit board during the FPC process to increase the coupling efficiency. The current stimulation threshold of microelectrodes in stroke rehabilitation is higher than the 30 µC·cm^−2^ recommended limit [37], therefore, we recommend that commercial IPGs need to be custom-designed to support this new/upcoming indication.

Recent evidence indicates that ECS plays a necessary auxiliary treatment role for limb function rehabilitation [13] after stroke, although the mechanisms underlying cortical stimulation (CS) remain unknown. ECS techniques have been reported in neuropathic pain [38], visual restoration, and tinnitus therapy [39]. Most interpretations suggest that enhanced neuroplasticity with more robust long-term learning and reorganization of neural circuits, possibly mediated by Gamma amino butyric acid (GABA)-ergic intracortical networks, may contribute to the improved neuronal functions associated with ECS [13]. It is believed that excitation of the motor cortex also results in inhibition of nociceptive neurons in somatosensory areas [38]. An implanted system should enhance efficacy and perhaps result in longer-term functional improvement [32].

Our implantable epidural interface showed application potential for cortical stimulation. The epidural interface can be implanted on the motor cortex to deliver electrical stimulation for limb rehabilitation in stroke survivors or for aphasia therapy, because it is less invasive than intracortical stimulation. Moreover, the wireless passive scheme is suitable for long-term modulation of neuronal motor function, auxiliary detoxification, and certain psychiatric disorders. Our scheme will be helpful to researchers who study the unknown mechanisms underlying cortical stimulation as it relates to neuroplasticity. Our current focus is to develop a prototype as a proof-of-concept. Future studies are needed to explore multichannel IPGs that form complex stimulation patterns and provide high voltage/current stimuli to large areas of the cortex for motor rehabilitation, and to optimize the device and test the long-term therapeutic effects in a stroke model to judge whether the IPG meets the electrical stimulation requirements for visual impairment conditions.

## 4. Materials and Methods

### 4.1. System Schematic

**Figure 4:**
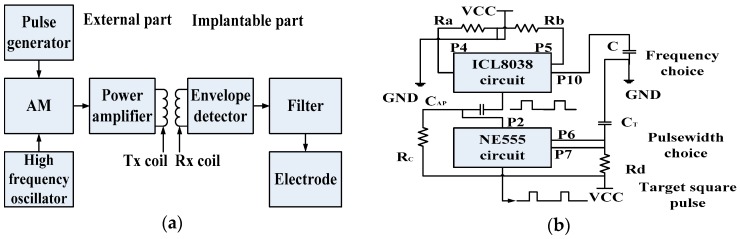

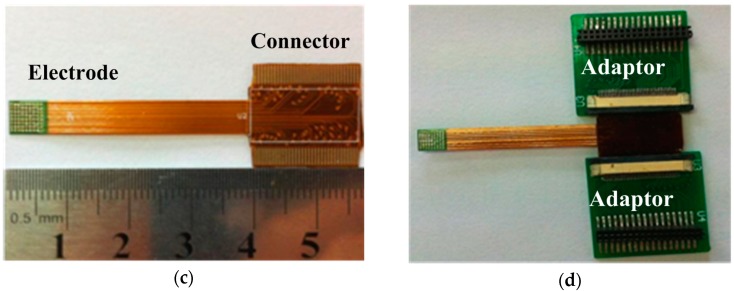
(**a**) The principle diagram of implantable single-channel stimulation system. The external part includes a pulse generation and transmission circuit array, and the implanted part includes a microcoil, demodulated circuit, and surface electrodes; (**b**) The schematics of the two-stage pulse generator circuit. The first stage circuit using ICL8038 is responsible for target frequency adjustment. The second stage circuit using NE555 is responsible for target pulse width regulation; Supply voltage (“VCC”); Ground (“GND”); (**c**) The surface micro-electrode array manufactured by six-layer flexible printed circuit (FPC) technique. The implantable part included 72 microelectrodes in about 5 × 5 mm^2^ area. The connecter part was connected to an independent external stimulator. It was finally abandoned due to high stiffness; (**d**) The electrode array has two bigger adaptor boards to connect the external stimulator. It was finally abandoned due to the size of the adaptor.

**Figure 5:**
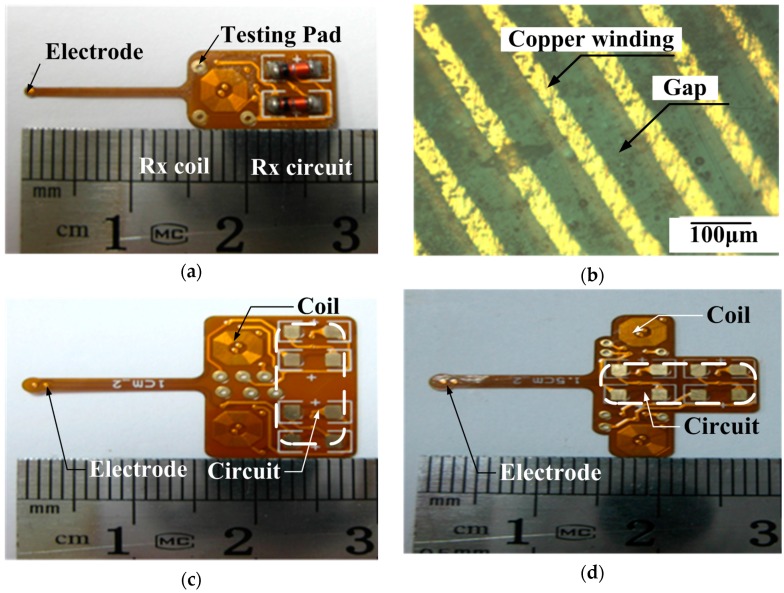
(**a**) Photograph of the single channel stimulator board; (**b**) Microscope image of the four-layer FPC coil (local). The four-layer FPC coils have a line width and spacing of 50 µm and a thickness of 17.5 µm; (**c**) A two-channel IPG interface with 10mm coils center distance; (**d**) A two-channel IPG interface with 15 mm coils center distance.

The proposed implantable neural interface comprises of an external and implanted part. As seen in Figure 4a, the external part includes a signal generator, carrier oscillator, amplitude modulation (AM) circuit, and power amplifier circuit supplied by regulated direct-current (DC) power supplies. We developed the implant interface with the external part modified from a previously described setup [30]. The implanted part includes a microcoil, passive demodulated circuit, and surface electrodes (Figure 5a). We chose 1.8 and 2 MHz as carrier frequencies in consideration of human safety. The passive demodulated circuit included a double-diode peak envelope detector and low-pass filter for demodulating the low frequency stimulus signal. We designed a two-stage signal generator circuit in the transmitter module to replace the NE555 circuit used in the previous version [30]. As shown in Figure 4b, the first stage used an ICL8038 waveform generation, which was responsible for adjusting target frequency, and a NE555, which was responsible for target pulse width. This two-stage adjustment design can improve the adjustment of pulse width and frequency of the square pulse voltage at the transmitter side.

The electrode used in the original version was a six-layer FPC 72-electrode array in a 5 × 5 mm^2^ area for high electrode density (Figure 4c,d), but it had high stiffness due to its 0.33 mm thickness and large adaptor board, making this electrode not suitable for epidural stimulation. Therefore, we fabricated a softer surface electrode suitable for epidural stimulation with only 0.17 mm thickness (Table 2) using a four-layer FPC technique, with gold immersion of 100 nm to strengthen the electrode electrochemical performance.

As shown in Figure 5a,b, we fabricated the receiving coil with octagonal spiral shape, L2, with 52 turns (4.2 mm in diameter) by four-layer FPC with 70 µm total thickness to couple with the external PCB microcoil L1 with 25 turns (8.4 mm diameter). Both line width and line spacing of the copper micro coil were 50 µm with 17.5 µm thickness (Figure 5b). The substrate layer and insulation layer, made of polyimide (PI) with 50 µm thickness, was biocompatible for acute animal experiment. The weld holes on the chip were for demodulated signal testing. The single channel receiver board interface with an area of 12 mm × 8 mm, except for the surface of the stimulating electrode, was insulated by a circuit insulation adhesive that was non-toxic, transparent, and made of acrylic acid and rubber. The integration of three parts including coil, demodulated circuit, and stimulating electrode makes the implantation into the epidural space more convenient than the previous design, which had separate electrodes and IPG. This new integration provides a more stable physical connection for stimuli delivery than the previous welded connection.

Two versions of the two-channel implantable interface were developed, with microcoil center distances (*R*_0_) of 10 and 15 mm, respectively (Figure 5c,d). Two versions were developed because the signal coupling coefficient in the target channel reached the maximum value when *R*_0_ was 15 mm, while the noise coupling coefficient in the target channel reached the minimal value when *R*_0_ was 10 mm. Both implantable interfaces needed an external microcoil array with the same *R*_0_ to inductively couple.

#### Benchtop Testing

Coil characteristics and impedance of the stimulating electrode were tested as described previously [40] using a series network analyzer (Agilent Technologies E5061A, Santa Clara, CA, USA) and an impedance/gain phase analyzer (1260A, Solartron Analytical, Farnborough, UK). The wireless transmission experimental testing platform was built using a transmitting printed circuit board (PCB) coil and a receiving four-layer FPC coil attached to annular rings to maintain a steady coupling distance. As the input voltage of the oscillator circuit (*V*_oc_) and the input voltage of the power amplifier circuit (*V*_pc_) at the transmitter circuit were changed, the output *V*_o_ of the implant board was recorded at 0.5, 1 and 2 Hz [41,42] with less than 1 ms pulse width at 2 mm coupling axial distance. The output *V*_o_ at the implant board was tested three times. The simulation of heat level on head tissue was completed under a carrier of 2 MHz and 10 V amplitude using Pennes bio-heat transferring equation.

### 4.2. Anesthesia and Surgery

The experiment protocol was carried out with approval from the Chinese Third Military Medical University Animal Care and Use Committee. Five normally sighted adult cats (1.9–2.5 kg) were used. We checked the refractive quality of eyes, light sensitivity, and verified that the cats had normal eyesight and that the V1 had normal physiological excitability. The cats were initially anesthetized with Sumianxin II (compound of xylidinothiazoline, Ethylenediaminetetraacetic acid (EDTA), dihydroetorphine hydrochloride, and haloperidol) (0.4 mL/kg, IM). Five to ten minutes later, the cats were given injections of atropine sulfate (0.3 mL, SC) to minimize gland secretion, and dexamethasone phosphate (1.5 mg/kg, IM) to minimize cerebral edema. Anesthesia was induced with urethane (20 mg/kg/h, IV) using a micro pump. Muscle paralysis was required to reduce Electromyography (EMG) artifact and achieved using Gallamine Triethiodide (10 mg/kg/h, IV). A water heating pad was set to body temperature. After intubation, a ventilator was used with tidal volume (TV) at 13.53 mL/kg and frequency at 20 breaths per minute. The oxygen saturation of the cat was measured during the experiment to make sure it was higher than 95%. The surgery methods used have been previously described [43]. Each cat was placed prone on a stereotactic frame (SR-6N, Narishige, Japan). A unilateral area (4 mm × 4 mm) above Areas 17 (V1, primary visual cortex) and 18 (V2) of the cat’s nearside hemisphere [44] and a contralateral 1 mm × 1 mm area above V1 were exposed by craniotomy. The experiment was conducted in a quiet and homothermal room. The cat’s cardiac rate and rectal temperature were continuously monitored during the experiment. All experiments were strictly carried out in accordance with the Experimental Animal Management Regulations of China National Science and Technology Council. At the end of the experiment, the cats were euthanized with intravenous injection of 30% urethane.

### 4.3. Stimulation and Recording Paradigms

As shown in Figure 3a–c, the stimulating electrode, fabricated by FPC technique, was placed on the unilateral dura mater above areas 17 (V1, primary visual cortex) and 18 (V2) of the cat [44]. The recording electrode was implanted in the contralateral V1. The stimulating electrode and recording electrode were always located in the same position of the bilateral visual cortex. Contralateral recording was conducted in order to avoid surface transmission of electrical stimuli. The response on the contralateral V1 was recorded using a multi-channel physiological acquisition and processing system (Chengdu Instrument Factory, Chengdu, China) after ECS in unilateral V1 was delivered. A micromanipulator and stereotactic frame were used for electrode insertion. To verify the efficacy of the epidural interface, sub-threshold electrical stimulation to the ipsilateral V1 and recording at the electrode-dura interface was also conducted by choosing a monophasic voltage pulse stimulus with low amplitude that was less than 1 V and pulse width less than 1 ms, as shown in Figure 3d. The custom-designed wired stimulator used a data acquisition card (compactDAQ9172) and USB-virtual instrument (NI 9265) (National Instrument co. ltd, Austin, TX, USA).

The receiving circuit was fixed on a shallow hole of the skull next to the stimulating electrode window. As shown in Figure 3e, an annular wall of 2 mm height was built on the surface of the receiving coil by sticking a plastic ring on the FPC coil by glue. The two leads of the implantable board were output voltage and ground. We fixed the receiving coil onto the transmitting coil’s array board in order to maintain the coupling distance. Figure 3f shows the two-channel implantable board with annular rings. Stainless steel needle electrodes in the scalp and in the nasal root acted as a reference and a ground. Each cat’s eyes were blinded using a black cloth in a quiet darkroom, as described in [41].

All evoked cortical responses were trigger-averaged over 30 repeated trials. The sampling rate for cortico-cortical evoked potential (CCEPs) was 2 KHz while filter bandwidth was between 0.16 Hz and 500 Hz throughout this study. CCEPs were analyzed after averaging the signals and filtering 50 Hz using MATLAB software (version R2009b). We compared the local field potential (LFP) waveform in contralateral visual cortex, before and after electrical stimulation, and calculated the average value of the amplitude and latency of CCEP at different stimulation frequency. As seen in Figure 3c, the epidural stimulation paradigm was less invasive and reduced local reactions by maintaining the integrity of the dura mater.

## 5. Conclusions

An implantable neuronal interface including a pulse generator and stimulating electrode was developed and evaluated in benchtop studies and in vivo. The benchtop hardware testing proved that the output of the pulse generator was reliably controlled by the input voltage of the transmitter. Our implantable neuronal interface demonstrated feasibility of the stimulator in a cat acute experiment. CCEPs were recorded on the contralateral visual cortex, which was comparable to those using wired stimulators in other stimulation-recording paradigms. Both benchtop testing and the cat experiment verified the validity of our implantable pulse generator. The implantable pulse generator showed application potential in ECS due to circuit flexibility and the integrity.

## Figures and Tables

**Table 1:** The output testing of pulse generator.

TX	*V*_pc_/V	5	6	7	8	9	10	11	12
RX	*V*_o1_/mV	100	200	300	300	350	250	400	430
	*V*_o2_/mV	500	600	700	900	1000	1200	1600	1650
	*V*_o3_/mV	250	400	350	500	600	700	1000	1000
	*V*_o_/mV	283	400	450	566	650	717	1000	1027
TX	*V*_pc_*/*V	12.9	13.9	14.9	16	17	18	19	20
RX	*V*_o1_/mV	450	500	520	500	550	600	700	750
	*V*_o2_/mV	1600	1600	2100	2200	2300	1500	2300	2600
	*V*_o3_/mV	950	650	930	1000	1500	1500	1600	1500
	*V*_o_/mV	1000	917	1183	1233	1450	1200	1533	1617

The output amplitude of pulse generator varied with the input voltage of power amplifier circuit (*V*_pc_) at transmitter side; the frequency was 2 Hz, pulse width was 0.5 ms, coupling axial distance was 2 mm, and load resistance was 1 KΩ. The output was tested three times when *V*_oc_ was 5 V. *V*_o_ = (1/3) × (*V*_o1_ + *V*_o2_ + *V*_o3_). Key: Transmitter side (“TX”); Receiver side (“RX”).

**Table 2:** The thickness of flexible printed board (FPC).

FPC Board	Thickness (mm)				Soft Level
	Electrode Center	Electrode Periphery	Wire	Circuit/Connect	
Six-layer	0.16	0.14	0.10	0.33	More rigid
Four-layer	0.16	0.14	0.04	0.17	More soft

## Abbreviations

CCEP	Cortico-Cortical Evoked Potential
ECS	Epidural Cortical Stimulation
IPG	Implantable Pulse Generator
FPC	Flexible Printed Circuit
